# Proposal of new diagnostic criteria for fatal familial insomnia

**DOI:** 10.1007/s00415-022-11135-6

**Published:** 2022-05-03

**Authors:** Min Chu, Kexin Xie, Jing Zhang, Zhongyun Chen, Imad Ghorayeb, Sven Rupprecht, Anthony T. Reder, Arturo Garay, Hiroyuki Honda, Masao Nagayama, Qi Shi, Shuqin Zhan, Haitian Nan, Jiatang Zhang, Hongzhi Guan, Li Cui, Yanjun Guo, Pedro Rosa-Neto, Serge Gauthier, Jiawei Wang, Xiaoping Dong, Liyong Wu

**Affiliations:** 1 Department of Neurology, Xuanwu Hospital, Capital Medical University, Beijing, 100053 China; 2 Institut de Neurosciences Cognitives et Intégratives d’Aquitaine, UMR 5287, Université de Bordeaux, 33076 Bordeaux, France; 3 CNRS, Institut de Neurosciences Cognitives et Intégratives d’Aquitaine, UMR 5287, 33076 Bordeaux, France; 4 Département de Neurophysiologie Clinique, Pôle Neurosciences Cliniques, CHU de Bordeaux, 33076 Bordeaux, France; 5 Hans Berger Department of Neurology, Jena University Hospital, Erlanger Alle 101, Jena, Germany; 6 Department of Neurology, University of Chicago, Chicago, IL USA; 7 Medicina del Sueño-Neurología-Centro de Educación Médica e Investigaciones Clínicas “Norberto Quirno” (CEMIC), Ciudad de Buenos Aires, Argentina; 8 Department of Neuropathology, Graduate School of Medical Sciences, Kyushu University, 3-1-1 Maidashi, Higashi-ku, Fukuoka, Japan; 9 Department of Neurology, International University of Health and Welfare (IUHW) Graduate School of Medicine, 4-3 Kozunomori, Narita, Chiba Japan; 10 State Key Laboratory for Infectious Disease Prevention and Control, National Institute for Viral Disease Control and Prevention, Chinese Center for Disease Control and Prevention, Beijing, 102206 China; 11 University of Yamanashi Kofu, Yamanashi, Japan; 12 Department of Neurology, General Hospital of the People’s Liberation Army, Beijing, China; 13 Department of Neurology, Peking Union Medical College Hospital, Chinese Academy of Medical Sciences and Peking Union Medical College, Beijing, China; 14 Department of Neurology, the First Hospital of Jilin University, Jilin University, Changchun, China; 15 Department of Neurology, Beijing Tongren Hospital, Capital Medical University, Beijing, China; 16 Alzheimer’s Disease Research Unit, McGill Centre for Studies in Aging, Montreal, Canada; 17 National Clinical Research Center for Geriatric Disease, Beijing, China

**Keywords:** Fatal familial insomnia, Prion disease, Diagnosis

## Abstract

**Background:**

The understanding of fatal familial insomnia (FFI), a rare neurodegenerative autosomal dominant prion disease, has improved in recent years as more cases were reported. This work aimed to propose new diagnostic criteria for FFI with optimal sensitivity, specificity, and likelihood ratio.

**Methods:**

An international group of experts was established and 128 genetically confirmed FFI cases and 281 non-FFI prion disease controls are enrolled in the validation process. The new criteria were proposed based on the following steps with two-round expert consultation: (1) Validation of the 2018 FFI criteria. (2) Diagnostic item selection according to statistical analysis and expert consensus. (3) Validation of the new criteria.

**Results:**

The 2018 criteria for possible FFI had a sensitivity of 90.6%, a specificity of 83.3%, with a positive likelihood ratio (PLR) of 5.43, and a negative likelihood ratio (NLR) of 0.11; and the probable FFI criteria had a sensitivity of 83.6%, specificity of 92.9%, with a PLR of 11.77, and a NLR of 0.18. The new criteria included more specific and/or common clinical features, two exclusion items, and summarized a precise and flexible diagnostic hierarchy. The new criteria for possible FFI had therefore reached a better sensitivity and specificity (92.2% and 96.1%, respectively), a PLR of 23.64 and a NLR of 0.08, whereas the probable FFI criteria showed a sensitivity of 90.6%, a specificity of 98.2%, with a PLR of 50.33 and a NLR of 0.095.

**Conclusions:**

We propose new clinical diagnostic criteria for FFI, for a better refining of the clinical hallmarks of the disease that ultimately would help an early recognition of FFI and a better differentiation from other prion diseases.

**Supplementary Information:**

The online version contains supplementary material available at 10.1007/s00415-022-11135-6.

## Introduction

Human prion diseases are a group of fatal progressive neurodegenerative disorders with various manifestations caused by the presence of scrapie-like prion protein, and encompass Creutzfeldt–Jakob disease (CJD), fatal familial insomnia (FFI), and Gerstmann–Sträussler–Scheinker syndrome (GSS) [1–3]. FFI is an autosomal dominant inherited disease, characterized by sleep-related, neuropsychiatric, and progressive sympathetic symptoms, with genetic analysis as a gold standard [4–6]. However, clinically early recognition and diagnosis of FFI remains difficult because of the incomplete penetrance, high clinical heterogeneity, no specificity of auxiliary examinations, and overlapping clinical profile with other prion diseases such as CJD and GSS [7, 8]. In addition, FFI is quite rare, so the vast majority of neurologists have seen few or no cases. Thus, precise clinical diagnostic criteria are critical for an early recognition of FFI.

FFI was first identified in a 1986 post-mortem examination [9]; however, the proposal of a clinical diagnostic pathway for FFI was delayed until 2014 [10]. In 2018, our institution published the clinical diagnostic criteria for FFI that were based on core clinical, suggestive and diagnostic features [8]. The core clinical features are probable organic sleep-related symptoms, rapidly progressive dementia (RPD) and progressive sympathetic symptoms. However, as the criteria were based on examination of a limited number of cases, their applicability to the recognition of FFI and its differentiation from other types of prion disease is unclear. Validation in additional samples and revision is needed to improve their diagnostic power.

In this study, we established a task force composed of internationals experts experienced in prion diseases to update the FFI diagnostic criteria based on the 2018 FFI diagnostic criteria. A modified Delphi methods and web-based interactive conferences approach were used to suit the specific needs of this task force.

## Materials and methods

### Ethics

This study in human subjects was approved by the institutional review board of Xuanwu Hospital. Written informed consent was obtained from all participants (or their guardians) before study initiation. Protocols were performed under the relevant guidelines and regulations for the use of human subjects in research set by the Chinese government. Clinical data of all participants were identified in this study.

### Procedure for validating and updating diagnostic criteria

To generate the updated diagnostic criteria, an international expert group was established that included experts in neurology, epidemiology, neuroimage, sleep medicine, neuropathology and neurophysiology from Europe, North America, South America and Asia. The experts are assigned to different groups according to their expertise, one group to define video PSG features (Imad Ghorayeb, Sven Rupprecht, Shuqin Zhan and Jiawei Wang), one for definition of dysautonomia (Anthony T Reder, Arturo Garay, Qi Shi and Xiaoping Dong), one for definition of cognitive functions (Pedro Rosa-Neto, Serge Gauthier and Liyong Wu), one group for neuroimage and neuropathology (Hiroyuki Honda, Masao Nagayama, Hongzhi Guan and Li Cui), one group for genetic test (Haitian Nan, Yanjun Guo and Jiatang Zhang). The updated criteria were generated in a three-step approach.

First, we searched PubMed, Embase, Wanfang, Sinomed, and CNKI databases from the first publication of the FFI in 1986 to September 2020 using the search terms shown in Supplementary Table S1. A systematic search and pooled analysis of the literature were carried out by two investigators (JZ and MC). Cases that met the CJD criteria diagnosed as possible or probable CJD were enrolled in our study [11]. All patients completed PRNP genetic analysis, gCJD patients had *PRNP* gene mutations while sCJD patients did not [12]. As no GSS diagnostic criteria have been established, these cases were diagnosed based on typical clinical symptoms, family history, auxiliary examinations, and genetic analysis.

The identified references for all cases included in the present analysis are listed in Supplementary Tables S2, S3, and S4. FFI patients with the *PRNP* D178N–129M mutation and gCJD and GSS patients with sufficient information from a case report or series were included in the analysis. Duplicate patients, those without genetic results or sufficient information, gCJD patients with the *PRNP* D178N–129M mutation, and individuals diagnosed with sporadic fatal insomnia were excluded.

Article selections were independently conducted by two authors (KXX and MC). Discrepancies in study selection and quality assessment were resolved by rechecking the source articles and through discussion with a third author (LYW) until a consensus was reached.

Second, we collected the case series reported in our institution. A total of 11 FFI patients were consecutively enrolled at the Department of Neurology of Xuanwu Hospital from 2012 to 2021, diagnosed with definite FFI with a prion protein (*PRNP*) gene mutation. The control group comprised patients with other types of prion disease (sporadic [s]CJD, *n* = 114; genetic [g]CJD, *n* = 5; and GSS, *n* = 2) who were consecutively enrolled at the Department of Neurology of Xuanwu Hospital from 2012 to 2021.

The subjects included in the validation process were patients recruited in our institution and those selected from published literature. We selected cases with sufficient clinical ratings, one of the three supportive ratings (documented family history, complete video polysomnography (video PSG), or PET/ SPECT, and EEG or MRI data for the further validation process). Ultimately, 128 FFI, 106 sCJD, 104 gCJD, and 71 GSS patients were included in the final validation analysis.

The following information was extracted from the identified articles and patients’ medical records by neurologists with expertise in prion diseases: sex, age of disease onset, disease duration, definitive family history, three clusters of clinical symptoms (sleep-related, neuropsychiatric, and progressive sympathetic), and results of five auxiliary examinations (genetic analyses, brain MRI, EEG, video PSG, and PET/SPECT).

Third, based on the evidence obtained in the first two steps, we validated the 2018 FFI criteria. The sensitivity of the diagnostic criteria published in 2018 (shown in Supplementary Table S5) was evaluated in FFI cases; they were then applied to CJD and GSS patients to assess their specificity. The validation process was divided into possible and probable FFI categories. Patients with probable organic sleep-related symptoms in addition to one or two other core features were diagnosed as possible FFI; and those who met the criteria for possible FFI diagnosis with a positive family history of rapidly progressive dementia (RPD) and insomnia or who had video PSG/PET/SPECT abnormalities were diagnosed as probable FFI. The sensitivity, specificity, and positive and negative likelihood ratios (PLR and NLR, respectively) were calculated.

The members in the expert group independently provided written feedback to the process coordinator (LYW), and then integrate the suggestions into the optimized criteria through two modified Delphi rounds. In July 2021, the expert group convened for a web-based consensus meeting to discuss the validation process and all aspects of the updated criteria (hierarchy diagnostic structure, core features, supportive features, definite features, exclusion criteria and candidate diagnostic items). The detailed information of the candidate items is described in the Result part. After the consensus meeting, we compared the frequency of candidate clinical, supportive, and exclusion features between FFI cases and controls, and selected discriminative and more prevalent items in FFI groups as diagnostic items, and those that rarely occurred in FFI but were frequently observed in controls as exclusion items. A final set of diagnostic criteria was proposed when the optimal PLR and NLR were achieved (PLR > 10 and NLR < 0.1) [13]. After revising and validating the new criteria, the written manuscript was circulated again and optimized in further modified Delphi rounds, particularly for accurate wording, appropriate definition, and flexible application. After final approval, the current criteria version was drafted and circulated to each member in expert group to reach final consensus.

### Statistical analysis

SPSS v23.0 software (IBM, Armonk, NY, USA) was used for all statistical analyses. The *χ*^*2*^ test and two-sample Student’s *t* test were used to assess between-group differences in categorical and continuous variables, respectively. Sensitivity was defined as the proportion of possible FFI or probable FFI cases among total FFI cases. Specificity was defined as the proportion of patients who did not meet the criteria for FFI in the total number of control cases. PLR was calculated as sensitivity/(1−specificity) and NLR as (1−sensitivity)/specificity. Differences were considered statistically significant at *p *< 0.05.

## Results

### Patient characteristics

Detailed demographic and clinical data of the study population are summarized in Table 1. No significant difference was found in sex ratio. The median age of FFI onset was 49 years (range 17–76 years), which was younger than sCJD and gCJD. The mean duration of FFI was 11 months (range 4–48 months), which was longer than sCJD (median 8 months; range 3–20) and shorter than GSS (median 48 months; range 3–156). In patients with FFI, prevalence of sleep-related symptoms, neuropsychiatric symptoms and progressive sympathetic symptoms were high (89.8%, 99.2% and 74.2%, respectively). The prevalence of sleep-related and progressive sympathetic symptoms differed significantly between FFI and other prion diseases, while neuropsychiatric symptoms had similar prevalence across the four groups.

**Table 1:** Demographic and clinical characteristics of the study population

		Control (non-FFI patients, *n* = 281)		
	FFI (*n* = 128)	sCJD (*n* = 106)	gCJD (*n* = 104)	GSS (*n* = 71)
Sex (F/M)	56/72	48/58	49/55	31/40
Age of onset, years	49 (17, 76)	61.5 (29, 83)*	49.5 (24, 92)*	46 (27, 74)
Disease duration, months	11 (4, 48)	8 (3,20)*	11 (2, 84)	48 (3, 156)*
Definite family history (*n*/total)	104/120	0*	78/104*	60/68
Sleep-related symptoms	115 (89.8%)	29 (27.4%)*	19 (18.3%)*	3 (4.2%)*
Neuropsychiatric symptoms	127 (99.2%)	100 (100%)	104 (100%)	71 (100%)
Progressive sympathetic symptoms	95 (74.2%)	10 (9.30%)*	7 (6.70%)*	3 (4.2%)*

Data are shown as median (range) or *n* (%) unless otherwise indicated

*F* female, *FFI* fatal familial insomnia, *gCJD* genetic Creutzfeldt–Jakob disease, *GSS* Gerstmann–Sträussler–Scheinker disease, *M* male, *sCJD* sporadic Creutzfeldt–Jakob disease

*p < 0.05 (sCJD, gCJD, and GSS vs FFI)

### Validation of 2018 diagnostic criteria

**Fig. 1:**
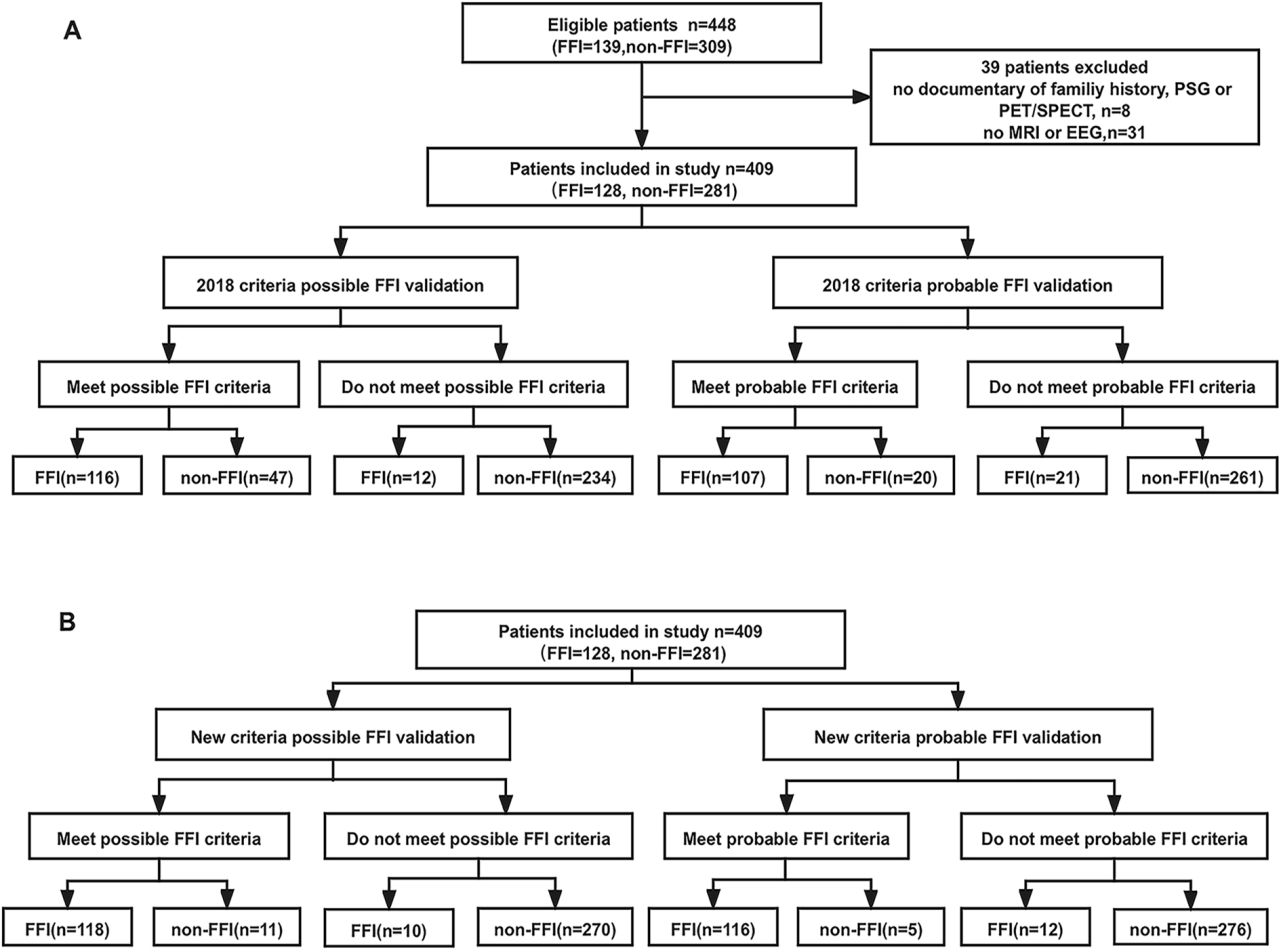
Case selection and validation process of the FFI diagnostic criteria. **a** 2018 FFI diagnostic criteria. **b** new FFI diagnostic criteria

The flow chart of case selection and validation of the 2018 criteria was shown in Fig. 1A.

### Possible FFI

According to the existing (2018) criteria, 116 cases were diagnosed as possible FFI, corresponding to a sensitivity of 90.6% (Table 2). Among the controls, 234 cases did not meet the diagnostic criteria for possible FFI, corresponding to a specificity of 83.3%. The PLR and NLR were 5.43 and 0.11. The criteria for possible FFI had a specificity of 73.6%, 82.7%, and 98.6% in differentiating sCJD, gCJD, and GSS, respectively.

**Table 2:** Validation of possible FFI according to 2018 FFI diagnostic criteria

	FFI (*n* = 128)	sCJD (*n* = 106)	gCJD (*n* = 104)	GSS (*n* = 71)	Control (*n* = 281)
Possible FFI/not FFI	116/12	28/78	18/86	1/70	47/234
Sensitivity	90.6%				
Specificity		73.6%	82.7%	98.6%	83.3%
PLR		3.43	5.24	60.71	5.43
NLR		0.13	0.11	0.095	0.11

*FFI* fatal familial insomnia, *gCJD* genetic Creutzfeldt–Jakob disease, *GSS* Gerstmann–Sträussler–Scheinker disease, *NLR* negative likelihood ratio *PLR* positive likelihood ratio, *sCJD* sporadic Creutzfeldt–Jakob disease

### Probable FFI

According to the existing criteria, 107 cases were diagnosed as probable FFI, corresponding to a sensitivity of 83.6% (Table 3). Among the controls, 261 cases did not meet the diagnostic criteria for probable FFI, corresponding to a specificity of 92.9%. The PLR is 11.77 and NLR is 0.18. The criteria for probable FFI had a specificity of 97.2% in differentiating sCJD, 84.6% in gCJD, and 98.6% in GSS.

**Table 3:** Validation of probable FFI according to 2018 FFI diagnostic criteria

	FFI (*n* = 128)	sCJD (*n* = 106)	gCJD (*n* = 104)	GSS (*n* = 71)	Control (*n* = 281)
Probable FFI/not FFI	107/21	3/103	16/88	1/70	20/261
Sensitivity	83.6%				
Specificity		97.2%	84.6%	98.6%	92.9%
PLR		29.7	5.43	59.71	11.77
NLR		0.17	0.19	0.17	0.18

*FFI* fatal familial insomnia, *gCJD* genetic Creutzfeldt–Jakob disease, *GSS* Gerstmann–Sträussler–Scheinker disease, *NLR* negative likelihood ratio *PLR* positive likelihood ratio, *sCJD* sporadic Creutzfeldt–Jakob disease

### Diagnostic item selection

As the sensitivity and specificity of the 2018 criteria did not meet our prespecified optimal thresholds, we concluded that revisions were necessary. This involved revising existing items and adding exclusion items.

Based on the recent literature and collective empirical knowledge of the experts, agrypnia excitata, neuro-ophthalmologic dysfunction, weight loss, and hyperthermia were suggested as potential items to incorporate into the revised diagnostic criteria for FFI. The term agrypnia excitata (agrypnia meaning “chasing sleep away” referring to sleep loss of organic origin, and excitata referring to the motor and autonomic activation) defines a generalized overactivation syndrome characterized by severe and persistent insomnia and marked motor and autonomic sympathetic activation (including weight loss and hyperthermia) [14]. Oneiric stupor is described as a peculiar behavior of agrypnia excitata, with the recurrence of stereotyped gestures mimicking simple daily life activities associated with the reporting of a dream mentation consisting in a single oneiric scene [15, 16]. Video PSG objective measures of agrypnia/sleep loss typically show reduction or disappearance of slow waves sleep, disappearance of spindles and the persistence of stage 1 non-rapid eye movement (NREM) sleep while rapid eye movement (REM) sleep, when identified, may persist but fails to stabilize, appearing in short recurrent episodes, either isolated, or mixed with stage 1 NREM sleep [14, 17]. In many FFI cases, sleep stages including REM sleep are difficult to identify and sleep scoring according to the AASM criteria [17] is difficult to apply because of loss or disruption of baseline electrophysiological brain activity and sleep specific features (spindles, K-complexes and REM sleep muscle atonia). Neuro-ophthalmologic dysfunction includes visual disturbances and ocular motor disorders. Neuropsychiatric symptoms are also common in prion and other neurogenerative diseases and are thus not useful for the recognition and differentiation of FFI. We formulated a detailed definition that included three main symptoms—namely, hallucination, delusion, and personality change/abnormal behavior.

To improve specificity, we added exclusion criteria. The relatively low specificity of the 2018 criteria was mainly attributed to the misdiagnosis of 26.4% of sCJD patients and 17.3% of gCJD patients as possible FFI. Thus, some criteria were deemed necessary to help exclude these CJD patients. By comparing the diagnostic criteria for CJD and FFI, we found it difficult to make a clear differentiation based on clinical symptoms. We therefore selected biomarkers from more sensitive auxiliary examination methods in the tests part of CJD including periodic sharp wave complex (PSWC) on EEG; hyperintensities in the caudate nucleus and putamen or at least two cortical regions (temporal–parietal–occipital) on diffusion-weighted imaging (DWI) or fluid-attenuated inversion recovery (FLAIR) MRI; and positive detection of 14-3-3 protein in cerebral spinal fluid (CSF).

The occurrence frequency of each candidate diagnostic item is shown in Table 4. Sleep-related symptoms such as probable organic insomnia, agrypnia excitata, sleep-related involuntary movement, sleep-related breathing disturbances, and laryngeal stridor were more common in FFI than in CJD and GSS and were mostly specific to FFI and were therefore emphasized in the new criteria. Sleep-related involuntary movements are defined as hypnic jerks, restless sleep, myoclonus, tremor, and twitchy non-purposeful movement of limbs. Sleep-related disturbances of breathing control were defined as sleep-related apnea/hypopnea syndrome.

**Table 4:** Frequency of diagnostic items between FFI and non-FFI groups

	FFI (*n* = 128)	sCJD (*n* = 106)	gCJD (*n* = 104)	GSS (*n* = 71)
Clinical features				
Probable organic sleep-related symptoms				
Probable Organic insomnia	111 (86.7%)	28 (25.9%)*	18 (17.3%)*	3 (4.2%)*
Agrypnia excitata	87 (71.9%)	0*	4 (3.8%)*	0*
Sleep-related involuntary movement	58 (45.3%)	20 (18.5%)*	2 (1.9%)*	0*
Sleep-related breathing disturbances (apnea/dyspnea)	33 (25.7%)	0*	2 (1.9%)*	0*
Laryngeal stridor	43 (33.5%)	3 (2.8%)*	1 (1.0%)*	0*
Neuropsychiatric symptoms				
Rapidly progressive dementia	108 (84.4%)	105 (99.1%)*	96 (92.3%)	49 (69.9%)*
Psychiatric symptoms	87 (68.0%)	41 (38.7%)*	55 (52.9%)*	23 (31.2%)*
Hallucination	44 (34.4%)	30 (28.3%)	23 (22.1%)	1 (1.1%)*
Delusion	32 (25.0%)	16 (15.1%)	15 (14.4%)	1 (1.1%)*
Personality change/abnormal behavior	87 (68.0%)	26 (24.5%)*	37 (35.6%)*	17 (19.4%)*
Abnormalities involving specific brain areas				
Cerebellar dysfunction	73 (57.0%)	56 (52.8%)	59 (56.7%)	64 (94.6%)*
Pyramidal dysfunction	28 (21.9%)	43 (40.6%)*	26 (25.0%)	21 (26.9%)
Extrapyramidal dysfunction	45 (35.2%)	51 (48.1%)*	41 (39.4%)	8 (14.0%)*
Neuro-ophthalmological dysfunction	34 (26.6%)	29 (27.3%)	19 (18.3%)	28 (38.7%)*
Progressive sympathetic symptoms				
Hypertension	43 (33.6%)	0*	2 (1.90%)*	1 (1.1%)*
Tachycardia	31 (24.2%)	0*	0*	0*
Irregular breathing	21 (16.4%)	0*	2 (1.90%)*	0*
Hyperthermia	27 (21.1%)	2 (1.9%)*	3 (2.90%)*	0*
Sweating	75 (58.5%)	2 (1.9%)*	2 (1.90%)*	0*
Weight loss	49 (38.2%)	5 (4.7%)*	3 (2.90%)*	2 (2.2%)*
Supportive features				
Positive family history of probable organic insomnia related symptoms	104/120	0/106*	7/104*	0/68*
Video PSG abnormality	56/56	4/8*	2/4*	2/6*
PET/SPECT positive	41/49	6/14*	2/11*	7/19*
Exclusion features				
Periodic sharp wave complex	3/100	30/105*	35/98*	4/54
DWI or FLAIR positive	1/94	88/106*	39/59*	17/63*
CSF 14-3-3 protein-positive	20/58	41/102	27/36*	6/18

*DWI* diffusion-weighted imaging, *FFI* fatal familial insomnia, *FLAIR* fluid-attenuated inversion recovery, *gCJD* genetic Creutzfeldt–Jakob disease, *GSS* Gerstmann–Sträussler–Scheinker disease, *PSG* polysomnography, *sCJD* sporadic Creutzfeldt–Jakob disease, *SPECT* single-photon emission computed tomography

*p < 0.05 (sCJD, gCJD, and GSS vs FFI)

Psychiatric symptoms including hallucination, delusion, and personality change/behavioral abnormality were also observed at a higher frequency in FFI than in the other diseases and were added to the revised diagnostic criteria. Cerebellar, pyramidal, extrapyramidal, and neuro-ophthalmologic dysfunction were more common in GSS and CJD than in FFI and were therefore discarded. Last, progressive sympathetic hypersensitivity symptoms were specific to FFI, especially sweating and weight loss. Abnormal vital signs including hypertension, tachycardia, irregular breathing, and hyperthermia were frequently observed in FFI patients but rarely reported in CJD and GSS. As these symptoms are convenient to evaluate in clinical settings, we thought they were highly recommended for inclusion in the revised diagnostic criteria.

Given that about 13.3% of FFI patients did not have predominant probable organic insomnia and thus failed to meet the criteria for possible FFI, there were some cases of missed diagnosis with the 2018 criteria. To cover as many of these patients as possible under the new criteria, we adjusted the weight of clinical features so that a diagnosis of possible FFI was made if patients had two out of three clinical features. Finally, based on our finding that median disease duration in FFI patients was 11 months, we defined the disease course as generally < 2 years to distinguish between the rapid and progressive course of FFI and insidious onset and slow progression of other neurodegenerative diseases.

PSWCs on EEG (FFI vs CJD, 3% vs 32.0%) and hyperintensities on DWI (FFI vs CJD, 1.1% vs 77.0%) were frequently observed in CJD but rarely in FFI and were therefore selected as candidate exclusion items. However, detection of 14-3-3 protein in CSF was not sufficiently rare in FFI to be considered as an exclusion criterion. We evaluated the sensitivity and specificity and determined PLR and NLR for the different combinations of MRI and EEG features to achieve the optimal cut-offs of sensitivity > 90%, specificity > 90%, PLR > 10, and NLR < 0.1 (Supplementary Tables S6 and S7). As the optimal diagnostic status was achieved with both set of features, they were included as exclusion items in the new diagnostic criteria (Table 5).

**Table 5:** New diagnostic criteria for FFI

Core clinical features with duration generally < 2 years
(a) Probable organic sleep-related symptoms: intractable insomnia, agrypnia excitata, accompanied with/without laryngeal stridor, sleep related apnea/hypopnea, and/or involuntary movements
(b) Neuropsychiatric symptoms: rapidly progressive dementia, psychiatric symptoms including hallucination, delusion, personality change and behavior abnormality
(c) Progressive sympathetic symptoms: hypertension, tachycardia, irregular breathing, hyperthermia, sweating, and/or weight loss
Supportive features
Positive family history of probable organic insomnia related symptoms
Probable organic insomnia (loss of circadian rhythm,sleep fragmentation, reduction of total sleep time, sleep–wake cycle disruption with an early permanent and progressive reduction in spindles, K complexes, delta waves and slow wave sleep) accompanied with/without sleep-related apnea/hypopnea, laryngeal stridor, and involuntary movements revealed by video polysomnography
Selectively low glucose uptake or hypoperfusion in the thalamus by PET or SPECT imaging
Exclusion features
(a) Periodic sharp wave complex on EEG
(b) Hyperintense signal in caudate nucleus and putamen or at least two cortical regions (temporal–parietal–occipital) in DWI or FLAIR sequences
(c) Pattern of deficits can be explained by other medical disorders
Diagnostic features
* PRNP* gene D178N-129M mutation
Diagnosis using the criteria
Possible FFI: Showing two out of three core clinical features without exclusion features
Probable FFI: Fulfill criteria for possible FFI + 1 or more suggestive features without exclusion features
Definite FFI: Fulfill two out of three core clinical features + diagnostic features

Probable organic insomnia related symptoms were emphasized, defined as rapidly progressive, intractable, and resistant to sedative drugs, with fragmentation and deterioration of sleep architecture. Sleep-related involuntary movements were defined as hypnic jerks, restless sleep with frequent changes in body position, and twitchy non-purposeful movement of limbs. Sleep-related symptoms also included oneiric stupor, status dissociatus, rapid eye movement sleep behavior disorder, pseudosleep, and arousal-related motor behavioral episodes (AMBEs). RPDs are neurologic conditions that develop subacutely over weeks to months; sympathetic symptoms were new manifestations accompanying disease progression; and weight loss was defined as a weight reduction of > 10 kg in the prior 6 months. Some nonspecific psychiatric symptoms including depression, anxiety, apathy, and confusion were also seen in early FFI

*DWI* diffusion-weighted imaging, *EEG* electroencephalogram, *FFI* fatal familial insomnia, *FLAIR* fluid-attenuated inversion recovery, *PET* positron emission computed tomography, *PRNP* prion protein, *SPECT* single-photon emission computed tomography

### Validation of the new diagnostic criteria

The flow chart of case selection and validation of the new criteria was shown in Fig. 1B.

### Possible FFI

A total of 118 FFI cases were diagnosed as possible FFI, yielding an increased sensitivity of 92.2% compared to the 2018 criteria (Table 6). A total of 270 CJD and GSS cases were excluded from a diagnosis of possible FFI, resulting in an increase in specificity to 96.1%. The PLR (23.64) and NLR (0.08) reached the optimal cut-off values. The new criteria had a specificity of 95.3%, 94.2%, and 100% in differentiating sCJD, gCJD, and GSS, respectively.

**Table 6:** Validation of possible FFI according to new FFI diagnostic criteria

	FFI (*n* = 128)	sCJD (*n* = 106)	gCJD (*n* = 104)	GSS (*n* = 71)	Control (*n* = 281)
Possible FFI/not FFI	118/10	5/101	6/98	0/70	11/270
Sensitivity	92.2%				
Specificity		95.3%	94.2%	100%	96.1%
PLR		19.64	15.91	−	23.64
NLR		0.08	0.08	0.08	0.08

*FFI* fatal familial insomnia, *gCJD* genetic Creutzfeldt–Jakob disease, *GSS* Gerstmann–Sträussler–Scheinker disease, *NLR* negative likelihood ratio, *PLR* positive likelihood ratio, *sCJD* sporadic Creutzfeldt–Jakob disease

### Probable FFI

One hundred and sixteen FFI cases that were diagnosed as probable FFI, which showed an increased sensitivity of 90.6% (Table 7). A total of 276 CJD and GSS cases were excluded from a diagnosis of probable FFI, which increased the diagnostic specificity to 98.2%. The PLR (50.33) and NLR (0.095) reached the optimal cut-off values. The criteria had a specificity of 98.1%, 97.1%, and 100% in differentiating sCJD, gCJD, and GSS, respectively.

**Table 7:** Validation of probable FFI according to new FFI diagnostic criteria

	FFI (*n* = 128)	sCJD (*n* = 106)	gCJD (*n* = 104)	GSS (*n* = 71)	Control (*n* = 281)
Probable FFI/not FFI	116/12	2/104	3/101	0/71	5/276
Sensitivity	90.6%				
Specificity		98.1%	97.1%	100%	98.2%
PLR		47.79	31.24	−	50.33
NLR		0.094	0.096	0.092	0.095

*FFI* fatal familial insomnia, *gCJD* genetic Creutzfeldt–Jakob disease, *GSS* Gerstmann–Sträussler–Scheinker disease, *NLR* negative likelihood ratio, *PLR* positive likelihood ratio, *sCJD* sporadic Creutzfeldt–Jakob disease

## Discussion

This study was an international effort to draft a new set of diagnostic criteria for FFI with improved sensitivity, specificity, and likelihood ratios through validation and revision of the 2018 criteria. The rigorous development process ensured that our findings are representative and generalizable. The new FFI criteria can aid the early diagnosis of FFI and help better differentiation from other prion diseases.

### New FFI diagnostic criteria

Some major changes are reflected in the new criteria. Firstly, a reliable diagnostic item selection procedure based on data analysis and modified Delphi method were used to identify the characteristic clinical features of FFI and minimize arbitrary definitions and ambiguity. Secondly, we added a definition for the general disease course of FFI, which is rapid and progressive. Thirdly, incorporation of EEG and/or MRI findings as an exclusion item was helpful for eliminating some CJD cases, which improved the specificity of the diagnostic criteria. Lastly, we included a clearer definition of family history that was restricted to probable organic insomnia-related symptoms.

We established a clear diagnostic hierarchy of possible, probable, and definite FFI depending on level of diagnostic and exclusionary certainty that can be easily implemented in clinical practice. Diagnosis of possible FFI is based solely on clinical features and can identify patients at the earliest stage of disease, based on the flexible combination of two of three clinically discriminating features—namely, sleep-related symptoms, neuropsychiatric symptoms, and progressive sympathetic symptoms. A diagnosis of probable FFI is based on clinical features plus a demonstrable family history of probable organic insomnia-related symptoms or video PSG or PET/SPECT markers that reflect objective functional impairment. Conversely, a diagnosis of FFI may be withheld if there are EEG and MRI or other biomarkers that are strongly indicative of CJD or other neurodegenerative diseases.

### Sensitivity and specificity of possible FFI

The sensitivity of possible FFI increased from 90.6% with the 2018 criteria to 92.2% with the new criteria, which was largely attributed to the new flexible structure of clinical signs where the presence of two out of three clinical features is sufficient for a diagnosis of possible FFI. This covers some FFI patients who do not have insomnia as a complaint. Compared to the 2018 diagnostic criteria for FFI, the specificity improved from 83.3 to 96.1% with the new criteria, indicating that the latter have better discriminatory power. This is mainly due to a more comprehensive framework with two items that excluded CJD and GSS cases that were previously identified as FFI based on 2018 criteria [18–25].

New items were also incorporated into the new criteria to help improve the sensitivity. Agrypnia excitata is a combination of loss of slow wave sleep (agrypnia) and autonomic and motor hyperactivation (excitata) that reflects sleep abnormalities in FFI [14, 26]. Besides FFI, laryngeal stridor is a clinical feature for multiple system atrophy (MSA) with a high diagnostic positive predictive value [27], however, this neurodegenerative disorder is characterized by slowly progressive autonomic failure, cerebellar ataxia, parkinsonism, that can easily be distinguished from the more rapidly progressive course of FFI.

In FFI, abnormal vital signs indicate cardiovascular dysfunction and can result from increased sympathetic activation; as they are easily evaluated quantitatively, are useful for monitoring clinical status [28]. Hyperthermia is frequently reported in FFI and mainly occurs early in the disease course without any signs of infection, which should be carefully defined because many patients have fever in their terminal phase when experiencing severe infection. Weight loss was the most frequent sympathetic symptom in FFI patients and has been previously defined with a cut-off point > 10 kg during last 6 months [10]. These symptoms, which are frequently observed in FFI, can facilitate early diagnosis.

Neuro-ophthalmologic findings were detected through analysis of video recordings of FFI patients within the previous 6 months; however, most neurologists are unaware of this manifestation, which likely biased the frequency estimation. Nonetheless, we tried to determine the incidence of neuro-ophthalmologic findings including visual disturbance and ocular motor disorders [29], although they were not included among the diagnostic items because they were nonspecific and easily confused with cerebellar symptoms.

### Sensitivity and specificity of probable FFI

The diagnostic items for probable FFI were limited to a demonstrable family history or positive video PSG/PET/SPECT signs and did not change drastically from the 2018 criteria. Nonetheless, the sensitivity of probable FFI increased from 83.6 to 90.6% with the update, which was mainly attributable to the improvement of possible FFI. The only changes in the new criteria for probable FFI from the previous version were the definition of family history, which was restricted to probable organic insomnia related symptoms, and the two specific exclusion items that increased the specificity from 92.9 to 98.2% by excluding many CJD cases.

### Prospects for clinical application

The new diagnostic criteria for FFI have good prospects for application in clinical settings with the increased sensitivity, specificity, and likelihood ratios. Firstly, video PSG and PET/SPECT are discriminative markers for the recognition of FFI. MRI and EEG contributed to the improved specificity and facilitated the discrimination of FFI from other prion diseases. Thus, we recommend the video PSG, PET/SPECT, MRI, and EEG examination to aid diagnosis of FFI if PRNP genetic analysis is not available. Secondly, we observed that patients with FFI had more obvious autonomic symptoms than those with other prion diseases, suggesting that these are characteristic features of FFI that can improve discrimination. Sensitive and objective autonomic biomarkers can be incorporated into future amendments of FFI diagnostic criteria. Lastly, some promising auxiliary examinations such as real-time quaking-induced conversion for detection of the abnormal form of prion protein in CSF and CSF total tau assessment may improve the clinical differentiation of prion diseases [30, 31].

## Limitations

Firstly, considering the rarity of this disease, it is unrealistic to build a large prospective cohort. The retrospective design and some of the cases identified in the literature may have introduced bias into our analyses, leading to an underestimation of actual symptom frequency. Secondly, as some auxiliary examinations were not performed in published studies, there may be some discrepancies in the positive rates for these assessments. Thirdly, neuropathology findings are the gold standard for sCJD diagnosis; however, biopsy or autopsy are difficult to achieve in real clinical practice. Last, we validated the specificity of diagnostic criteria in CJD and GSS, further validation needs to be conducted in other neurological diseases.

## Conclusions

A comprehensive, integrated, and concise diagnostic scheme for FFI was established. The new diagnostic criteria can aid early recognition of FFI and better discrimination from other prion diseases with optimal specificity and sensitivity and likelihood ratios, help effectively further screening and patient management, with a high value in clinical practice.

## Ethics approval

The clinical protocols were approved by the Ethics Committee and local Institutional Review Board of Xuanwu Hospital, Capital Medical University, China.

## Supplementary Information

Below is the link to the electronic supplementary material.

Supplementary file1 (DOCX 20 KB)

Supplementary file2 (PDF 97 KB)

Supplementary file3 (PDF 77 KB)

Supplementary file4 (PDF 83 KB)

## Data Availability

The data that support the findings of this study are available on request from the corresponding author. The data are not publicly available due to privacy or ethical restrictions.
