# Supplementary material for: Proposal of new diagnostic criteria for fatal familial insomnia
Source: J Neurol. 2022 May 3;269(9):4909–19. doi: 10.1007/s00415-022-11135-6 (PMC9363306; doi:10.1007/s00415-022-11135-6)
Supplement: Supplementary file 1 — Supplementary file1 (DOCX 20 KB) [file 415_2022_11135_MOESM1_ESM.docx]

**eTable 1 Search items ^a^**

| FFI | "Insomnia, Fatal Familial"[Mesh] OR "familial insomnia" OR "fatal insomnia" OR “FFI” OR ((familial insomnia OR fatal insomnia) AND (prion OR PRNP OR scrapie prion protein OR PrPSc OR PrPc OR PrP gene)). |
| --- | --- |
| CJD GSS | ((Familial OR Hereditary OR Inherited) AND ("Creutzfeldt-Jakob Syndrome"[Mesh] OR creutzfel* OR jakob* OR CJD)) OR (fCJD) for fCJD, and “Gerstmann-Straussler-Scheinker Disease” [Mesh] OR Gerstmann-Straussler-Scheinker OR gerstmann-sträussler-scheinker OR 'gerstmann straussler scheinker ' OR 'gerstmann sträussler scheinker' OR (gerstmann* AND (Straussler OR Sträussler OR Straussler Scheinker OR Scheinker)) OR (gerstmann* AND (prion OR PRNP OR scrapie prion protein OR PrPSc OR PrPc OR PrP gene)) for GSS |

^a^ Using PubMed Keywords as an example.

**eTable 2 the identified references for FFI cases included in the present analysis**

**eTable 3 the identified references for gCJD cases included in the present analysis**

**eTable 4 the identified references for GSS cases included in the present analysis**

**eTable 5 2018 Diagnostic criteria for FFI**

| **Core clinical features and possible fatal familial insomnia**  The organic sleep‑related abnormalities(a) in addition to one or two other core features (b/c) are essential for a diagnosis of possible FFI.  a. Organic sleep‑related symptoms: Insomnia, lack of deep sleep, sleep fragmentation and reduction or loss of REM sleep, laryngeal stridor, sleep breath disturbance, and involuntary movements;  b. RPD: The presence or absence of ataxia, pyramidal or extrapyramidal symptoms or signs, and psychiatric symptoms;  c. progressive sympathetic symptoms: Hypertension, sweating, tachycardia, and irregular breathing.  **Suggestive features and probable fatal familial insomnia**  If one or more of these suggestive features and two or more core features above are present, a diagnosis of probable FFI can be made.  a. Positive family history of RPD and insomnia  b. Organic insomnia, sleep‑related apnea, laryngeal stridor, and involuntary movements revealed by PSG  c. Low glucose uptake in the thalamus demonstrated by SPECT or PET imaging.  **Diagnostic features and definitive fatal familial insomnia**  If the *PRNP* gene test is positive, a diagnosis of definitive FFI can be confirmed.  *PRNP* gene sequencing revealed D178N mutation with methionine polymorphism at codon 129. |
| --- |

Abbreviations: FFI, fatal familial insomnia; PSG, polysomnography; RPD, rapidly progressive dementia; SPECT, single-photon emission computed tomography.

**eTable 6 Sensitivity, specificity and likelihood of Possible FFI in exclusion item selection process (Clinical feature a +b/c)**

| Clinical feature a +b/c | Sensitivity | Specificity | PLR | NLR |
| --- | --- | --- | --- | --- |
| PSWCs on EEG | 87.7% | 88.7% | 7.76 | 0.14 |
| High signal on MRI | 89.2% | 93.6% | 13.94 | 0.12 |
| PSWCs on EEG / high signal on MRI | 86.9% | 96.5% | 24.8 | 0.14 |
| No exclusion item | 90.6% | 83.3% | 5.43 | 0.11 |

Abbreviations: PSWC, periodic sharp wave complex; EEG, Electroencephalogram; MRI, magnetic resonance imaging; NLR, negative likelihood ratio PLR, positive likelihood ratio;

**eTable 7 Sensitivity, specificity, and likelihood of Possible FFI in exclusion item selection process [Clinical feature 2 out of 3 (a/b/c)]**

| Clinical feature 2 out of 3 (a/b/c) | Sensitivity | Specificity | PLR | NLR |
| --- | --- | --- | --- | --- |
| PSWCs on EEG | 94.6% | 86.5% | 7.00 | 0.06 |
| High signal on MRI | 93.1% | 95.7% | 21.65 | 0.07 |
| PSWCs on EEG / high signal on MRI | 92.2% | 96.1% | 23.64 | 0.08 |
| No exclusion item | 93.8% | 81.6% | 5.09 | 0.08 |

Abbreviations: PSWC, periodic sharp wave complex; EEG, Electroencephalogram; MRI, magnetic resonance imaging; NLR, negative likelihood ratio PLR, positive likelihood ratio;
