# Supplementary material for: Proposal of new diagnostic criteria for fatal familial insomnia
Source: J Neurol. 2022 May 3;269(9):4909–19. doi: 10.1007/s00415-022-11135-6 (PMC9363306; doi:10.1007/s00415-022-11135-6)
Supplement: Supplementary file 2 — Supplementary file2 (PDF 97 KB) [file 415_2022_11135_MOESM2_ESM.pdf]

Patient ID	First Author	Title	Journal	Nationality	Publish year
1	李华玲	家族性致死性失眠症的护理方法探讨	会议论文	China	2007
2	廉玲	睡眠障碍伴多系统改变的家族性致死性失眠症1例	中华全科医学	China	2019
3	侯乐	以精神症状为首发症状的家族性致死性失眠症状、影像及基因分析	会议论文	China	2013
4	陈彬	致死性家族性失眠症患者一例报告并文献复习	中国神经免疫学和神经病学杂志	China	2013
5~13	石琦	中国家族型致死性失眠症患者的 临床及家族特征分析	中国病毒病杂志	China	2012
14	彭彬	致死性家族性失眠症一例的临床、病理及基因特征	中华神经科杂志	China	2012
15	李悦	家族性致死性失眠1例报道	神经损伤与功能重建	China	2012
16~17	周珏倩	2例致死性家族性失眠症的临床特点、脑影像和朊蛋白基因分析	中国神经精神疾病杂志	China	2011
18	宋兴旺	广东省家族性致死性失眠症一家系临床特征及基因突变分析	中华神经医学杂志	China	2010
19	张敏	致死性家族性失眠症一例临床及基因特征	中华神经科杂志	China	2005
20-21	唐舒锦	致死性家族性失眠症临床表现及多导睡眠图特点分析	中国神经免疫学和神经病学杂志	China	2018
22	孙雅婷	初诊为额颞叶痴呆的家族性致死性失眠一例并文献复习	中华神经科杂志	China	2018
23~24	边洋	致死性家族性失眠症2家系患者的临床、影像及基因改变特点	中华医学杂志	China	2018
25	王湘庆	家族性致死性失眠症睡眠异常活动与EEG表现	会议论文	China	2015
26	卢婷婷	伴脑白质异常信号的家族性致死性失眠症一例	中华神经科杂志	China	2015
27~33	Runcheng He	Clinical features and genetic characteristics of two Chinese pedigrees with fatal family insomnia	PRION	China	2019
34	Congcong Sun	Agrypnia excitata and obstructive apnea in a patient with fatal familial insomnia from China A case report	Medicine	China	2017
35	Lin Sun	Familial fatal insomnia with atypical clinical features in a patient with D178N mutation and homozygosity for Met at codon 129 of the prion protein gene	Prion	China	2015
37	Wu-Ling Xie	Comparison of the pathologic and pathogenic features in six different regions of postmortem brains of three patients with fatal familial insomnia	INTERNATIONAL JOURNAL OF MOLECULAR MEDICINE	China	2013
38~39	Xiao-Hong Shi	Clinical, histopathological and genetic studies in a family with fatal familial insomnia	Infection, Genetics and Evolution	China	2010

40~41	Sian D. Spacey	Fatal Familial Insomnia<br>The First Account in a Family of Chinese Descent	Arch Neurol	China	2004
42	T.H. Yeh, W.J. Hong	Familial fatal insomnia: A Taiwanese case report	Journal of the Neurological Sciences	China	2019
42~52	patients in XUANWU				
53~54	Takuya Fukuoka	Clinical significance of fatal laryngeal stridor in fatal familial insomnia	Neurol Clin Neurosci.	Japan	2019
55	Mi Ji Lee	Midbrain Hypometabolism in Fatal Familial Insomnia: A Case Report and a Statistical Parametric Mapping Analysis of a Korean Family	Case Rep Neurol	korean	2014
56	Prashanth L. Kukkle	A Case of Autosomal Dominant Ataxia with Vocal Cord Palsy Attributed to a Mutation in the PRNP Gene	Movement Disorders	India	2020
57	E. Toribio-Ďiaz	Fatal familial insomnia: A new case description with early response to immunotherapy	Journal of Neuroimmunology	Spain	2020
58-59	Y. Saitoh	DISCORDANT CLINICOPATHOLOGIC PHENOTYPES IN A JAPANESE KINDRED OF FATAL FAMILIAL INSOMNIA	Neurology	Japan	2010
60	K. Sasaki	Fatal familial insomnia with an unusual prion protein deposition pattern: an autopsy report with an experimental transmission study	Neuropathology and Applied Neurobiology	Japan	2005
61	Joyce Schenkein	Self-management of Fatal Familial Insomnia. Part 2: Case Report	MedGenMed	US	2006
62	José Luiz Pedroso	COMPLEX MOVEMENT DISORDERS IN FATAL FAMILIAL INSOMNIA: A CLINICAL AND GENETIC DISCUSSION	Neurology	Brazil	2013
63	Jessica M Stevens	Case of fatal familial insomnia caused by a d178n mutation with phenotypic similarity to Hashimoto's encephalopathy	BMJ Case Rep	US	2018
64	Leonardo Cruz de Souza	Sexual disinhibition and agrypnia excitata in fatal familial insomnia	Journal of the Neurological Sciences	Italy	2016
65-66	P. Silburn, FRACP	Fatal familial insomnia:A seventh family	Neurology	US	1996
67-68	Mahlon D. Johnson	Fatal familial insomnia: Clinical and pathologic heterogeneity in genetic half brothers	Neurology	US	1998
69-72	P. Cortelli	Cerebral metabolism in fatal familial insomnia: Relation to duration, neuropathology, and distribution of protease-resistent prion protein	Neurology	US	1996
73	G. Rossi	Fatal familial insomnia<br>Genetic, neuropathologic, and biochemical study of a patient from a new Italian kindred	Neurology	Italy	1998
74-78	G. Almer	Fatal familial insomnia: a new Austrian family	Brain	Austria	1999

79-81	Anja Harder	Novel Twelve-Generation Kindred of Fatal Familial Insomnia From Germany Representing the Entire Spectrum of Disease Expression	American Journal of Medical Genetics	Germany	1999
82~83	C Tabernero	Fatal familial insomnia: clinical, neuropathological, and genetic description of a Spanish family	J Neurol Neurosurg Psychiatry	Spain	2000
84	Karl-Jürgen,Bär	Serial Positron Emission Tomographic Findings in an Atypical Presentation of Fatal Familal Insomnia	Arch Neurol	Germany	2002
85-86	Julia´n Benito-Leo´n	Combined Quinacrine and Chlorpromazine Therapy in Fatal Familial Insomnia	Clin Neuropharmacol	Spain	2004
87	A. Harder	Early age of onset in fatal familial insomnia Two novel cases and review of the literature	J Neurol	Germany	2004
88~99	J J Zarranz	Phenotypic variability in familial prion diseases due to the D178N mutation	J Neurol Neurosurg Psychiatry	Spain	2005
100	D. Dimitri	Fatal familial insomnia presenting as psychosis in an 18-year-old man	Neurology	France	2006
101	Marc Wermke	Frontal diaschisis in a German case of fatal familial insomnia	J Neurol	Germany	2006
102	Iriarte	Agrypnia excitata in fatal familial insomia.A VIDEO-polygraphic study	Neurology	Spain	2007
103	Hak,S	In Vivo Detection of Thalamic Gliosis<br>A Pathoradiologic Demonstration in Familial Fatal Insomnia	Arch Neurol	France	2008
104	Rita J.Guerreiro	A case of Demantia with PRNP D178Ncis-129M and No Insomnia	Alzheimer Dis Assoc Disord	Macedonia	2009
105	Alberto Raggi	The behavioural features of fatal familial insomnia: A new Italian case with pathological verification	Sleep Medicine	Italy	2009
106	Luis Fernando Casas-Mendez	Biot's Breathing in a Woman with Fatal Familial Insomnia: Is There a Role for Noninvasive Ventilation?	Journal of Clinical Sleep Medicine	Spain	2011
107	T. Froböse	Agomelatine Improves Sleep in a Patient with Fatal Familial Insomnia	Pharmacopsychiatry	Germany	2012
108	Angelo Gemignani	Thalamic contribution to Sleep Slow Oscillation features in humans: A single case cross sectional EEG study in Fatal Familial Insomnia	Sleep Medicine	Italy	2012
109	Sven Rupprecht	Does the Clinical Phenotype of Fatal Familial Insomnia Depend on PRNP codon 129 Methionine-Valine Polymorphism?	Journal of Clinical Sleep Medicine	Germany	2013
110-113	Elena Prieto	Metabolic patterns in prion diseases: an FDG PET voxel-based analysis	Eur J Nucl Med Mol Imaging	Spain	2015
114	Thomas Megelin	Fatal familial insomnia: a video-polysomnographic case report	Sleep Medicine	France	2017
115	Arturo Garay	The rhythms of AMBEs (arousal-related motor behavioral episodes) in Agrypnia Excitata: a video motor analysis	Sleep Medicine	Argentina	2019

116	Elisa Baldin	A case of fatal familial insomnia in Africa	J Neurol	Morocco	2009
117~119	D. Perani	[18F]FDG PET in fatal familial insomnia: The functional effects of thalamic lesions	Neurology	Italy	1993
120-121	Paul Brown	FFI Cases from the United States, Australia, and Japan	Brain Pathology	USA	1998
122	Robert G. Will	FFI Cases from the United Kingdom	Brain Pathology	UK	1998
123	C.A. McLean	The D178N (cis-129M)“fatalfamilial insomnia” mutation associated with diverse clinicopathologic phenotypes in an Australian kindred	Neurology	Austrilia	1997
124	Jos´e Eduardo E. Lima	Clinical Reasoning: A 45-year-old man with progressive insomnia and psychiatric and motor symptoms	Neurology	Brazil	2020
125-127	Jean Julien	The French FFI Cases	Brain Pathology	France	1998
128	Shuai Chen	Reduced cerebral blood flow in genetic prion disease with PRNP D178N–129M mutation: An arterial spin labeling MRI study	Journal of Clinical Neuroscience	China	2015
