# Supplementary material for: Proposal of new diagnostic criteria for fatal familial insomnia
Source: J Neurol. 2022 May 3;269(9):4909–19. doi: 10.1007/s00415-022-11135-6 (PMC9363306; doi:10.1007/s00415-022-11135-6)
Supplement: Supplementary file 3 — Supplementary file3 (PDF 77 KB) [file 415_2022_11135_MOESM3_ESM.pdf]

Patient ID	First Author	Title	Journal	Country	Publish year
1	E Mitrova	A case of Creutzfeldt-Jakob disease related to familial retinitis pigmentosa patients	European journal of epidemiology	Czechoslovakia	1988
2	Yooseok Kwon	A Case of Familial CJD With A PRNP E200K Mutation	poster	South Korea	
3	Sandrine Larue	A Case of Familial Creutzfeldt-Jakob Disease Presenting with Dry Cough	THE CANADIAN JOURNAL OF NEUROLOGICAL SCIENCES	Canada	2005
4	Ján Necpál	A Corticobasal Syndrome Variant of Familial Creutzfeldt-Jakob Disease with Stroke-Like Onset	Case Reports in Neurological Medicine	Slovakia	2016
5	Effrosyni Koutsouraki	A probable role of copper in the comorbidity in Wilson's and Creutzfeldt-Jakob's Diseases: a case report	Virology Journal	Greece	2020
6	Waliszewska-Prosół M	Cerebellar ataxia as a first manifestation of Creutzfeldt-Jakob disease in two cousins	Postgrad Med J	Poland	2018
7	Waleska Berrios	Familial CREUTZFELD JACOB DISEASE: A Case Report	Poster	Argentina	2018
8	John M. Bertoni	Familial Creutzfeldt-Jakob disease (codon 200 mutation) with supranuclear palsy	JAMA	Gemany	1992
9	Carlton S. Gass	Familial Creutzfeldt-Jakob Disease: A Neuropsychological Case Study	Archives of Clinical Neuropsychology	USA	2000
10	D. Kaul	Familial Creutzfeldt-Jakob disease: A novel phenotype associated with E200K-129M mutation	poster	USA	2015
11	Nancy Huang	Familial Creutzfeldt-Jakob disease associated with a point mutation at codon 210 of the prion protein gene	Arq Neuropsiquiatr	Brazil	2001
12~13	Y.Harigaya	Familial Creutzfeldt-Jakob disease carrying a V180I mutation with a MV129 polymorphism	J Neurol	Italy	2012
14	Kristin Clift	Familial Creutzfeldt-Jakob Disease: Case report and role of genetic counseling in post mortem testing	Prion	USA	2016
15	Nishit Sawal	Familial Creutzfeldt–Jakob Disease: The First Reported Kindred from South-East Asia	Annals of Indian Academy of Neurology	India	2019

16	C Yanagihara	Rapidly progressive dementia syndrome associated with a novel four extra repeat mutation in the prion protein gene	Journal of Neurology, Neurosurgery, and Psychiatry	Japan	2001
17	Kosuke Matsuzono	Single Photon Emission Computed Tomography (SPECT) Findings of a Patient with a Novel Prion Mutation	Internal Medicine	Japan	2014
18-19	G.P.A RICE	Spongiform Encephalopathy of Long Duration: A Family Study	LEJOURNAL CANADIEN DES SCIENCE NEUROLOGIQUES	Canada	1980
20	Kentaro Deguchi	Spreading brain lesions in a familial Creutzfeldt-Jakob disease with V180I mutation over 4 years	BMC Neurology	Japan	2012
21	I. Gandoglia	Stroke-like onset of Creutzfeldt-Jakob disease in a patient with the PRNP V203I mutation	European Journal of Neurology	Italy	2019
22	Cao Chen	The first Chinese case of Creutzfeldt-Jakob disease patient with R208H mutation in PRNP	Prion	China	2011
23	J. Chapman	Transmission of spongiform encephalopathy from a familial Creutzfeldt-Jakob disease patient of Jewish Libyan origin carrying the PRNP codon 200 mutation	Neurology	Israel	1991
24	Ghezzi A.	Two familial cases of Creutzfeldt-Jakob disease in Italy	The Italian Journal of Neurological Sciences	Italy	1989
25~26	Elisabeth Farbu	Two Norwegian sisters with late onset Creutzfeldt-Jakob disease caused by the E200K mutation	J Neurol	Norway	2006
27-28	Yu-Pu Guo	Virus-Induced Subacute Spongiform Encephalopathy (Creutzfeldt-Jakob Disease) A Report of Two Chinese Cases with Clinico-Pathologic Studies	Journal of the Neurological Sciences	China	1985
29	S.Papacostas	Ten-year mortality from Creutzfeldt-Jakob disease in Cyprus.	Eastern Mediterranean Health Journal	Cyprus	2008
30~31	Bart Dermaut	Familial Creutzfeldt-Jakob disease in a patient carrying both a presenilin 1 missense substitution and a prion protein gene insertion	J neurol	Belgium	2000
32	Sarosh M. Katrak	Familial Creutzfeldt-Jakob Disease in an Indian Kindred	Annals of Indian Academy of Neurology	India	2019
33~35	Takeshi Yamamoto	Familial Creutzfeldt-Jakob Disease in Japan	Journal of the Neurological Sciences	Japan	1985
35-38	E.Mitrova	Familial Creutzfeldt-Jakob disease with temporal and spatial separation of affected members	European journal of epidemiology	slovakia	1990
39	J.Candel	Familial Creutzfeldt-Jakob disease. All night v-polysomnography study	Clinical Neurophysiology	Spain	2016

40	David L Camenga	Familial Creutzfeldt-Jakob Disease	Annals new york academy of sciences	USA	
41	J. Chapman	Fatal insomnia in a case of familial Creutzfeldt-Jakob disease with the codon 200Lys mutation	Neurology	Israel	1996
42	Elison Sarapura-Castro	Familial Creutzfeldt-Jakob disease, with E200K mutation in Peru	poster	Lima-Peru	2017
43-44	Paola Imbriani	Heidenhain variant in two patients with inherited V210I Creutzfeldt–Jakob disease	International Journal of Neuroscience	Italy	2015
45~46	F.Caisberger	Heterogeneous clinical manifestation of familial Creutzfeldt-Jakob disease with D178N mutation	European Journal of Neurology	Czech Republic	2016
47	Jing Ye	Human prion disease with a G114V mutation and epidemiological studies in a Chinese family: a case series	Journal of Medical Case Reports	China	2008
48	Andre Granger	Hypertrophic Olivary Degeneration and Movement Disorder in a Patient with Familial Creutzfeldt-Jakob Disease	Cureus	USA	2020
49	Dnicholl	Inherited Creutzfeldt-Jakob disease in a British family associated with a novel 144 base pair insertion of the prion protein gene	Journal of Neurology, Neurosurgery, and Psychiatry	UK	2015
50	C Jansen	Inherited Creutzfeldt–Jakob disease in a Dutch patient with a novel five octapeptide repeat insertion and unusual cerebellar morphology	Journal of Neurology, Neurosurgery, and Psychiatry	Italy	2009
51	田杰	PNRP基因突变导致遗传型Creutzfeldt-Jakob病1例报告	家庭医药	China	2016
52	林世和	家族性Creutzfeldt_Jakob病_林世和	临床神经病学杂志	China	2007
53	王莉	遗传性朊蛋白病一家系的临床_病理和基因突变研究	国际数字医学会数字中医药分会成立大会暨首届数字中医药学术交流会	China	2016
54~55	S. A. Appel	Rapidly progressive Creutzfeldt–Jakob disease in patients with Familial Mediterranean Fever	European Journal of Neurology	Israel	2010
56	Shoudu Zeng	Psychotic Symptoms Presented in Familial Creutzfeldt-Jakob Disease, Subtype E200K	J Clin Psychiatry	USA	2001
57	John Collinge	Inheritedpriondisease(PrPlysine200)inBritain:two case reports	BMJ	uk	1993
58	A.L. Taratuto	Insomnia associated with thalamic involvement in E200K involvement in E200K	Neurology	Italy	2001
59~70	G.R.Mallucci	Inherited prion disease with an alanine to valine mutation at codon 117 in the prion protein gene	Brain	UK	1999

71~73	I.Inoue	Japanese family with Creutzfeldt- Jakob disease with codon 200 point mutation of the prion protein gene	Neurology	Japan	1994
74~76	Bo-Yeong Choi	Mutations at codons 178, 200-129, and 232 contributed to the inherited prion diseases in Korean patients	BMC Infectious Diseases	Republic of Korea	2009
77	Haruo Seno	New haplotype of familial Creutzfeldt-Jakob diseasewith a codon 200 mutation and a codon 219 polymorphism of the prion protein gene in a Japanese family	Acta Neuropathol	Japan	2000
78	Woei-Cherng Shyu	Panencephalitic Creutzfeldt-Jakob disease in a Chinese family Unusual presentation with PrP codon 210 mutation and identification by PCR-SSCP	Joural of the Neurological Sciences	China	1996
79	Maria Carmela Tartaglia	Pathologic Evidence That the T188R Mutation in PRNP Is Associated With Prion Disease	J Neuropathol Exp Neurol	USA	2010
80~82	Yuichi Hayashi	Preserved regional cerebral blood flow in the occipital cortices, brainstem, and cerebellum of patients with V180I-129M genetic Creutzfeldt-Jakob disease in serial SPECT studies	Journal of the Neurological Sciences	Japan	2016
83~87	Tomohiro Miyakawa	Japanese Creutzfeldt-jakob disease patients exhibiting high incidence of the E200K PRNP mutation and located in the basin of a river	Neurological Research	Japan	2017
88~96	Ricardo Nitrini	Prion Disease Resembling Frontotemporal Dementia and Parkinsonism Linked to Chromosome 17	Arq Neuropsiquiatr	Canada	2001
97-101	patients in Xuanwu hospital				
102-104	Cartier L	Familial clustering of the ataxicform of Creutzfeldt-Jakob disease with Hirano bodies	Journal of Neurology, Neurosurgery, and Psychiatry	USA	1984
