# Supplementary material for: Proposal of new diagnostic criteria for fatal familial insomnia
Source: J Neurol. 2022 May 3;269(9):4909–19. doi: 10.1007/s00415-022-11135-6 (PMC9363306; doi:10.1007/s00415-022-11135-6)
Supplement: Supplementary file 4 — Supplementary file4 (PDF 83 KB) [file 415_2022_11135_MOESM4_ESM.pdf]

Patient ID	First Author	Title	Journal	Country	Publish year
1~2	Sang-Beom Kim	Two Korean families with Gerstmann-Str€aussler-Scheinker disease	Prion 2015 Poster Abstracts	Republic of Korea	2015
3	Johann A. Hainfellner	The Original Gerstmann-Straussler-Scheinker Family of Austria: Divergent Clinicopathological Phenotypes but Constant PrP Genotype	Brain Pathology	Austria	1995
4~6	Ausrine Areskeviciute	Sporadic Creutzfeldt-Jakob Disease in a Woman Married Into a Gerstmann-Str€aussler-Scheinker Family: An Investigation of Prions Transmission via Microchimerism	J Neuropatho Exp Neurol	UK	2018
7~8	Federica Provini	Sleep and temperature rhythms in two sisters with P102L Gerstmann-Stra ̈ussler-Scheinker (GSS) disease	Sleep Medicine	Italy	2009
9	Yin Wang	Report on the first Chinese family with GerstmannSträussler-Scheinker disease manifesting the codon 102 mutation in the prion protein gene	Neuropathology	China	2006
10	Min Ju Kang	Pearls & Oy-sters: Challenging diagnosis of Gerstmann-Stra ̈ussler-Scheinker disease	Neurology	Republic of Korea	2019
11	G.Pierangeli	Normal sleep-wake and circadian rhythms in a case of GerstmannSträussler-Sheinker (GSS) disease	Clin Auton Res	Italy	2004
12	Hisako Furukawa	New variant prion protein in a Japanese family with Gerstmann-Straussler syndrome	Molecular Brain Research	Japan	1995
13-19	Ettore Salsano	Lower limb areflexia without central and peripheral conduction abnormalities is highly suggestive of Gerstmann–Sträussler–Scheinker disease Pro102Leu	Journal of the Neurological Sciences	Italy	2011
20	Robert Rusina	Gerstmann–Sträussler–Scheinker syndrome with the P102L pathogenic mutation presenting as familial Creutzfeldt–Jakob disease: a case report and review of the literature	Neurocase: The Neural Basis of Cognition	Czech Replublic	2014
21	John Woulfe	Gerstmann-Straussler-Scheinker disease with the Q217R mutation mimicking frontotemporal dementia	Acta Neuropatholo	Canada	2005
22	Marco Bianca	Gerstmann–Sträussler–Scheinker disease with P102L–V129 mutation:a case with psychiatric manifestations at onset	Annales de Genetique	Italy	2003
23~24	Arthur J.Hudson	Gersmann-Straussler-Scheinker Disease with coincidental Family Onset	Ann Neurol	Italy	1983
25	Sarah E Keuss	Gerstmann-Sträussler-Scheinker disease with atypical presentation	BMJ Case Rep	UK	2017
26	Andrew B.Fleming	Gerstmann-Sträussler-Scheinker Disease With a Progressive Supranuclear Palsy Presentation	Dement Geriatr Cogn Disord	USA	2010
27	Roser Ribosa-Nogue	Gerstmann-Straussler-Scheinker Disease Presenting with Atypical Parkinsonism, but Typical Magnetic Resonance Imaging Findings of Prion Disease	Movement Disorders	Spain	2015
28	L. Mumoli	Gerstmann–Straussler–Scheinker disease with PRNP P102L heterozygous mutation presenting as progressive myoclonus epilepsy	European Journal of Neurology	Italy	2017
29~30	K.Young	Gerstmann-Straussler-Scheinker disease with mutation at codon 102 and methionine at codon 129 of PRNP in previously unreported patients	Neurology	USA	1995

31	Nirav Bhatt	Gerstmann Straussler Scheinker Disease (GSS) with Rapidly Progressive Cognitive Decline After Head Trauma	American Nrurological Association	USA	2015
32	Yasushi Iwasaki	Gerstmann-Sträussler-Scheinker disease with P102L prion protein gene mutation presenting with rapidly progressive clinical course	Clinical Neuropathology	Japan	2014
33~34	Yamada,M	Involvement of the spinal posterior horn in Gerstmann-StrausslerScheinker disease (PrP P102L)	Neurology	Japan	1999
35~36	Constantine G.Lykesos	The Dementia of Gerstmann-Straussler Scheinker Syndrome:Clinical Variability Demonstrated by Two Case Reports	Journal of Neuropsychiatry	USA	1995
37	J.Collinge	Diagnosis of gerstmann-sträussler syndrome in familial dementia with prion protein gene analysis	The lancet	UK	1989
38~42	P.Brown	clinical and molecular genetic study of a large german kindred with Gerstmann-Straussler-Scheinker syndrome	Neurology	USA	1991
43	Min Jeong Park	A Case of Gerstmann-Sträussler-Scheinker Disease	J Clin Neurol	Korea	2010
44	A.Vital	A case of Gerstmann-Strussler-Scheinker disease with a novel six octapeptide repeat insertion	Neuropathology and Applied Neurobiology	France	2011
45	Yasushi Iwasaki	A case of Gerstmann-Sträussler-Scheinker syndrome with the P105L prion protein gene mutation presenting with ataxia and extrapyramidal signs without spastic paraparesis	Clinical Neurology and Neurosurgery	Japan	2009
46	Jing Wang	A Chinese patient of P102L Gerstmann-SträusslerScheinker disease contains three other diseaseassociated mutations in SYNE1	Prion	China	2018
47	Takanori Takazawa	A Distinct Phenotype of Leg Hyperreflexia in a Japanese Family with Gerstmann-Sträussler-Scheinker Syndrome (P102L)	Internal Medicine	Japan	2009
48	Ling Long	A family with hereditary cerebellar ataxia finally confirmed as Gerstmann-Sträussler-Scheinker syndrome with P102L mutation in PRNP gene	Neurosciences	China	2017
49~50	Yutaka Tanaka	A Japanese family with a variant of GerstmannStraiussler-Scheinker disease	Journal of Neurology, Neurosurgery, and Psychiatry	Japan	1997
51	Ainhoa Alzualde	A Novel PRNP Y218N mutation in gerstmann-strussler-scheinker disease with neurofibrillary degeneration	J neuropatho Exp Neurol	Spain	2010
52	Ayse Aralasmak	A prion disease - Possible gerstmann-straussler-scheinker disease A case report	J Comput Assist Tomogr	USA	2006
53	J.Fiala	First genetically proven case of Gerstmann-Straussler-Scheinker disease in the Czech Republic: a case report of an atypical clinical course	European Journal of Neurology	Czech Replublic	2010
54	Lev G.Goldfarb	An insert mutation in the chromosome 20 amyloid precursor gene in a Gerstmann-Striussler-Scheinker family	Journal of the Neurological Sciences	USA	1992

55	P.P.Liberski	A case of sporadic Creutzfeldt-Jakob disease with a Gerstmann-Sträussler-Scheinker phenotype but no alterations in the PRNP gene	Acta Neuropatholo	Poland	1998
56-58	Hong-Fu Li	Clinical features of Chinese patients with GerstmanneSträusslereScheinker identified by targeted next-generation sequencing	Neurobiology of Aging	China	2016
59~63	Michiyoshi Yoshimura	Correlation between clinical and radiologic features of patients with Gerstmann-Sträussler-Scheinker syndrome (Pro102Leu)	Journal of the Neurological Sciences	Japan	2018
64	M.Kojovic	De novo P102L mutation in a patient with Gerstmann-Stra¨ussler-Scheinker disease	European Journal of Neurology	UK	2011
65	Hiroyuki Honda	Different Complicated Brain Pathologies in Monozygotic Twins With Gerstmann–Str€aussler–Scheinker Disease	J Neuropatho Exp Neurol	Japan	2017
66	patient in xuanwu hospital				
67	靖冬来	PRNP基因P102L突变致非典型 Gerstmann-Sträussler-Scheinker 综合征一例并文献复习	中华神经科	China	2020
68~70	赵明明	Gerstmann-Sträussler-Scheinker病1例报告	中风与神经疾病杂志	China	2016
71	董海林	罕见遗传性共济失调的基因突变和临床表型特点	浙江大学硕士毕业论文	China	2018
